# Transient expression of full-length and mature nattokinase in Nicotiana benthamiana reveals early necrosis from full-length form and functional activity of the mature enzyme

**DOI:** 10.3389/fpls.2025.1631697

**Published:** 2025-07-21

**Authors:** Kevin Wang, Hugh Mason, Kylie Hall, Ethan Slone, Kylie Tackett, Nan Wang

**Affiliations:** ^1^ Division of Math and Natural Sciences, University of Pikeville, Pikeville, KY, United States; ^2^ School of Life Sciences, Arizona State University, Tempe, AZ, United States; ^3^ Biotechnology Research Institute, Chinese Academy of Agriculture Sciences, Beijing, China

**Keywords:** plant transient expression, codon optimization, nattokinase, BEYDV, Nicotiana benthamiana

## Abstract

Nattokinase is a potent fibrinolytic enzyme widely used in the treatment of cardiovascular diseases for its ability to directly degrade fibrin and plasmin substrates, effectively dissolving blood clots. In this study, both full-length and mature forms of the nattokinase coding sequence were transiently expressed in *Nicotiana benthamiana* using a modified Bean Yellow Dwarf Virus (BeYDV) replicon system. Overexpression of the full-length (pre-pro) construct resulted in severe leaf necrosis within 2.5 days post-infiltration (dpi), with electrolyte leakage analysis indicating an 83.5% loss of membrane integrity by 3 dpi. In contrast, the mature form of nattokinase was successfully expressed without early cytotoxicity and exhibited strong caseinolytic and fibrinolytic activity, reaching 22,500 FU/g—comparable to commercial standards. These findings demonstrate the feasibility of producing biologically active nattokinase in plants and highlight the potential of plant-based expression systems as scalable, cost-effective platforms for therapeutic enzyme production.

## Introduction

Cardiovascular diseases (CVDs) remain a leading cause of mortality worldwide, often due to complications arising from blood clot formation. Nattokinase, a serine protease produced by *Bacillus subtilis* during natto fermentation, has attracted significant interest for its potent fibrinolytic activity (Granito et al., 2024; Yang et al., 2024). It directly degrades fibrin and plasmin substrates and has shown therapeutic potential in the prevention and treatment of thrombosis, atherosclerosis, stroke, hypertension, and Alzheimer’s disease (Chiu et al., 2024; Jensen et al., 2016; Tanikawa et al., 2024). Nattokinase (NK) is currently under Phase 4 clinical evaluation for oral administration (e.g., NCT02886507, NCT02913170, NCT00447434), with no significant adverse effects reported to date.

Commercial nattokinase is primarily produced through *B. subtilis* fermentation; however, residual impurities in these preparations may trigger allergic reactions, raising regulatory and safety concerns. Recombinant production in alternative microbial hosts such as *Escherichia coli* and yeast has been explored (Ni et al., 2016; Jain et al., 2024; Yan et al., 2021), but these systems often suffer from limitations including inclusion body formation, low yields, and reliance on toxic or tightly regulated inducers—factors that constrain scalability and cost-effectiveness.

In contrast to conventional expression platforms, plant-based platforms have emerged as promising alternatives for recombinant protein production, offering key advantages in scalability, safety, and cost (Eidenberger et al., 2023; Buyel, 2024; Akher et al., 2025; Shanmugaraj et al., 2025). Studies have demonstrated the enzymatic activity of plant-derived nattokinase. For example, Han et al. (2015) transiently expressed a full-length, codon-optimized nattokinase gene in melon, achieving a fibrinolytic activity of 79.30 U/mL. Similarly, Ni et al. (2023) reported *in vivo* thrombolytic efficacy in transgenic cucumber plants harboring an integrated full-length nattokinase gene. Among available plant hosts, *Nicotiana benthamiana* is widely used for transient expression due to its rapid growth, well-characterized genome (Chen et al., 2024; Golubova et al., 2024; Ranawaka et al., 2024), and ability to accumulate high levels of recombinant protein within 4–7 days. Akher et al. (2025) review advancements in viral vector-mediated transient expression systems in *N. benthamiana*, emphasizing their central role in rapid, scalable biopharmaceutical production and integration with hydroponics and controlled environment agriculture to support regulatory compliance and meet market demands.

However, challenges remain. In plant expression systems, overexpression of precursor or zymogen forms of proteins—especially those containing native prokaryotic signal peptides or pro-domains—often leads to endoplasmic reticulum (ER) stress, unfolded protein response (UPR), and hypersensitive response (HR)-like symptoms (Buono et al., 2019; Chen et al., 2023). These stress responses can culminate in localized cell death or systemic necrosis, particularly when the foreign protein is not efficiently processed or misfolds in the ER. Studies have shown that transient expression of proteases in plants can induce pronounced tissue damage characterized by necrotic lesions and compromised cell viability, ultimately leading to cell death (Dickey et al., 2017; Ma et al., 2019). In this study, we employed a modified Bean Yellow Dwarf Virus (BeYDV) replicon system (Diamos and Mason, 2019) to transiently express both the full-length (pre-pro) and mature forms of nattokinase in *N. benthamiana.* Enzymatic activity was evaluated using casein and fibrin degradation assays, while tissue integrity was assessed through visual inspection and electrolyte leakage analysis. Our findings revealed that expression of the full-length nattokinase triggered severe leaf necrosis as early as 2.5 dpi, consistent with early onset of BeYDV-driven gene expression. These results underscore the cytotoxic risks associated with overexpression of full-length proteases in plant systems and highlight the importance of using mature, pre-processed protein constructs to enable safe, functional, and sustainable production of therapeutic enzymes in plant-based platforms. Nonetheless, to achieve the full cost-effectiveness and commercial viability of plant-derived nattokinase, several upstream and downstream challenges must be addressed. As highlighted by Buyel (2024), key bottlenecks include variable protein yield, batch-to-batch consistency, regulatory alignment, and scalable purification. A unified, technology-driven framework that integrates optimized expression, extraction, and purification strategies will be essential to translating plant molecular farming into a commercially viable production system for enzymes like nattokinase.

## Materials and methods

### Plant expression constructs and leaf agroinfiltration

To design the nattokinase (*aprN*) expression constructs, a murine tobacco mosaic virus-specific monoclonal antibody heavy chain signal peptide (LPH: MECNWILPFILSVTSGAYS; Hiatt et al., 1989; Voss et al., 1995; Vaquero et al., 1999; Schiermeyer et al., 2005; Knödler et al., 2023) was fused to the N-terminus of both the full-length and mature forms of the *aprN* protein from *B. subtilis* MTCC (GenBank accession number: KJ174339.1). A hexahistidine tag (His_6_) was added to the C-terminus to facilitate purification. These constructs, designated LPH-full-length-aprN-His_6_ and LPH-mature-aprN-His_6_, were codon-optimized for *N. benthamiana* using Invitrogen’s GeneOptimizer^®^ (Thermo Fisher Scientific, Waltham, MA, USA). The codon-optimized full-length *aprN* sequence has been deposited in GenBank under accession number PQ432224, which includes the signal peptide (residues 1–29), propeptide (residues 30–106), and the mature peptide (residues 107–381) (Weng et al., 2017).

**Figure 1:**
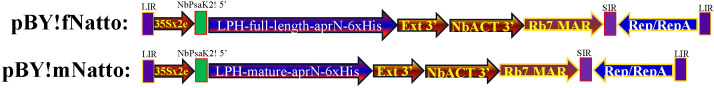
Schematic representation of the pBY!fNatto and pBY!mNatto expression vector for transient nattokinase expression in *N. benthamiana.* The plant expression vector pBY!fNatto (full-length nattokinase) and pBY!mNatto (mature-length nattokinase), utilized in this research, is based on the optimized BeYDV vector (Diamos and Mason, 2019) designed for high-level expression of a single target gene. The components of the vector include 35Sx2e, which is the 35S promoter from cauliflower mosaic virus enhanced with a duplicated enhancer region; NbPsaK2T! 5′, a truncated 5′ UTR from the *N. benthamiana* psaK gene; Ext 3′, a tobacco extensin terminator from which the intron has been removed; NbACT 3′, the 3′ UTR from the *N. benthamiana* ACT3 gene; Rb7 MAR, the tobacco Rb7 matrix attachment region; SIR, a short intergenic region from BeYDV; Rep/RepA, the replication proteins from BeYDV; and LIR, the long intergenic region from BeYDV. Additionally, the vector includes a plant codon-optimized murine monoclonal antibody heavy chain signal peptide (LPH) fused to the N-terminal of the codon-optimized nattokinase (aprN) sequence, with a hexahistidine (6xHis) tag at the C-terminal for Ni-NTA affinity purification.

To facilitate cloning, a XhoI–NbPsaK2!–5′–XbaI fragment was added to the 5′ end and a SacI site to the 3′ end of each construct. The complete cassettes—XhoI–NbPsaK2!–XbaI–LPH-full-length-aprN-His_6_–SacI and XhoI–NbPsaK2!–XbaI–LPH-mature-aprN-His_6_–SacI—were synthesized by Invitrogen’s GeneArt^®^ Gene Synthesis service and cloned into the XhoI and SacI sites of the pBYR2e-based vector (Diamos and Mason, 2019), generating the pBY!fNatto and pBY!mNatto constructs, respectively (**Figure 1**).

The plant expression vectors pBY!fNatto (full-length nattokinase) and pBY!mNatto (mature-length nattokinase) were validated by restriction enzyme digestion and PCR, then introduced into *Agrobacterium tumefaciens* strain EHA105 (GoldBio, St. Louis, MO, USA) via electroporation using a MicroPulser™ Electroporator (Bio-Rad, Hercules, CA, USA). Transformed *Agrobacterium* colonies were verified by PCR and plated on selective LB agar containing 50 mg/L kanamycin, followed by incubation at ~30°C for 3 days. For agroinfiltration, bacterial cultures were harvested and resuspended in infiltration buffer (10 mM 2-(N-morpholino) ethanesulfonic acid (MES), 10 mM MgSO_4_, pH 5.5) to an optical density at 600 nm (OD_600_) of 0.2. The suspension was used to infiltrate the abaxial surface of 6- to 10-week-old *N. benthamiana* leaves using a 20 mL syringe without a needle. Leaf samples were collected at 4 dpi for subsequent protein extraction and purification.

### Visualizing nattokinase-induced necrosis and electrolyte leakage assay

Tissue necrosis was assessed both visually and quantitatively using the electrolyte leakage (EL) assay, following the protocol described by Dickey et al. (2017). Leaf discs (5 mm diameter) were collected using a cork borer (5 mm diameter) from regions infiltrated with *A. tumefaciens* strain EHA105 alone, carrying either the pBY!fNatto or pBY!mNatto plasmid, or from untreated control leaves. On days 2.5, 3 and 4 post-infiltration (dpi), discs were briefly rinsed with deionized (DI) water to remove surface residues. Each disc was then placed into a 15 × 160 mm test tube containing 10 mL of DI water and incubated at room temperature on a shaker at 200 rpm for 1 hour. The initial conductivity (T_0_) was measured using a conductivity meter (Bante Instruments, Sugar Land, TX, USA).

Following T_0_ measurement, samples were boiled at 98°C for 2 hours in a water bath to release total electrolytes. After cooling, the volume was restored to 10 mL with DI water, and final conductivity (T_1_) was measured. The extent of electrolyte leakage (% EL), representing the degree of tissue damage, was calculated using the formula:


$$\%\text{ EL }=\;100\;\times \;\left({\text{T}}_{0}\;-\text{ DI}\right)/\left({\text{T}}_{1}\;-\text{ DI}\right)$$

where DI is the conductivity of the water control.

Results were expressed as means ± standard error (SE) for n = 3 biological replicates and statistically analyzed using pairwise Student’s t-tests.

### Protein analysis and characterization by SDS-PAGE and western blot

All materials, kits, and reagents used for SDS-PAGE and Western blotting were obtained from Thermo Fisher Scientific Inc. (Waltham, MA, USA), unless otherwise specified. Total soluble proteins were extracted from approximately 150 mg of leaf tissue—using the Pierce™ Plant Total Protein Extraction Kit, following the manufacturer’s protocol. Tissues were processed using either native lysis buffer supplemented with 0.1% Halt™ Protease Inhibitor Cocktail or denaturing lysis buffer, as appropriate for downstream applications.

For SDS-PAGE analysis, 20 µL of crude protein extract was mixed with 4X Bolt™ LDS Sample Buffer and 10X Bolt™ Reducing Agent. Deionized water was added to adjust the final volume to 40 µL. Samples were denatured at 70°C for 10 minutes prior to loading onto a Bolt™ Bis-Tris Plus Mini gel. Following electrophoresis, gels were stained with Imperial™ Protein Stain for visualization. His-tagged proteins were purified using HisPur™ Ni-NTA Spin Columns according to the manufacturer’s instructions. The eluted fractions were subsequently desalted using Zeba™ Micro Spin Desalting Columns (7K MWCO) and concentrated using Pierce™ Protein Concentrators (10K MWCO). Protein yield was estimated by analyzing band intensity with ImageJ software, using BSA standard bands as internal loading controls. Western blotting was performed using the SuperSignal™ West HisProbe™ Kit according to the supplied protocol. Detection was carried out using SuperSignal^®^ Working Solution, and the blots were imaged using a ChemiDoc™ XRS+ Imaging System (Bio-Rad, Hercules, CA, USA).

### Casein hydrolysis test and fibrinolytic activity assay

Protease activity of recombinant nattokinase (rNK) was initially assessed using a casein plate assay as described by Zhang et al. (2021). A 0.5% agarose gel containing 1% casein (Sigma-Aldrich, Allentown, PA, USA) was prepared in 1× phosphate-buffered saline (PBS) and poured into Petri plates to solidify. Wells (5 mm diameter) were created in the gel, and 3 µg of purified plant-derived rNK in 40 µL of 1× PBS was loaded into each well. Positive control wells received 3 µg of commercial nattokinase (AbMore BioScience Inc., Houston, TX, USA; Batch#: M2121-202401; Activity data: 22,500 FU/g), while negative control wells contained either PBS or crude supernatant from wild-type (non-infiltrated) leaves. Following a 5-hour incubation at 37°C, the gels were stained with Coomassie Brilliant Blue R-250 (Bio-Rad, Hercules, CA, USA) to visualize zones of casein degradation. Proteolytic activity was quantified by measuring the average of the long and short diameters of each clear zone, subtracting the original well diameter (5 mm). Results were expressed as means ± standard error (SE; *n* = 3) and statistical significance was evaluated using paired Student’s *t*-tests.

Fibrinolytic activity was further assessed using a modified fibrin plate assay following the protocol of Dickey et al. (2017). Briefly, 0.2 g of agarose was dissolved in 40 mL of 1× PBS by microwave heating and cooled to approximately 40°C. Before the gel solidified, 30 mg of human fibrinogen (Sigma-Aldrich), 8 units of human plasminogen (rPeptide LLC, Bogart, GA, USA), and 8 units of human thrombin (Medix Biochemica Inc., St. Louis, MO, USA) were added with gentle mixing to prevent bubble formation. The mixture was poured into Petri dishes and allowed to solidify at room temperature. Wells (5 mm diameter) were then created in the gel, and 5 µg of purified rNK diluted in 40 µL of 1× PBS was added to each well. Commercial nattokinase (5 µg; Abmole Bioscience Inc., Houston, TX, USA; Batch#: M2121-202401; Activity data:22,500 FU/g) was used as a positive control, while PBS and eluates from wild-type leaf extracts were included as negative controls. Plates were incubated overnight at 37°C. The diameters of the clear halos, with the well diameter subtracted, were measured to determine fibrinolytic activity. Enzymatic activity was quantified relative to the commercial standard (22,500 FU/g) using methods described in Dickey et al. (2017). Results were reported as means ± SE (*n* = 3) and analyzed using paired Student’s *t*-tests.

## Results

### Impact of full-length nattokinase expression on leaf necrosis and tissue integrity

**Figure 2:**
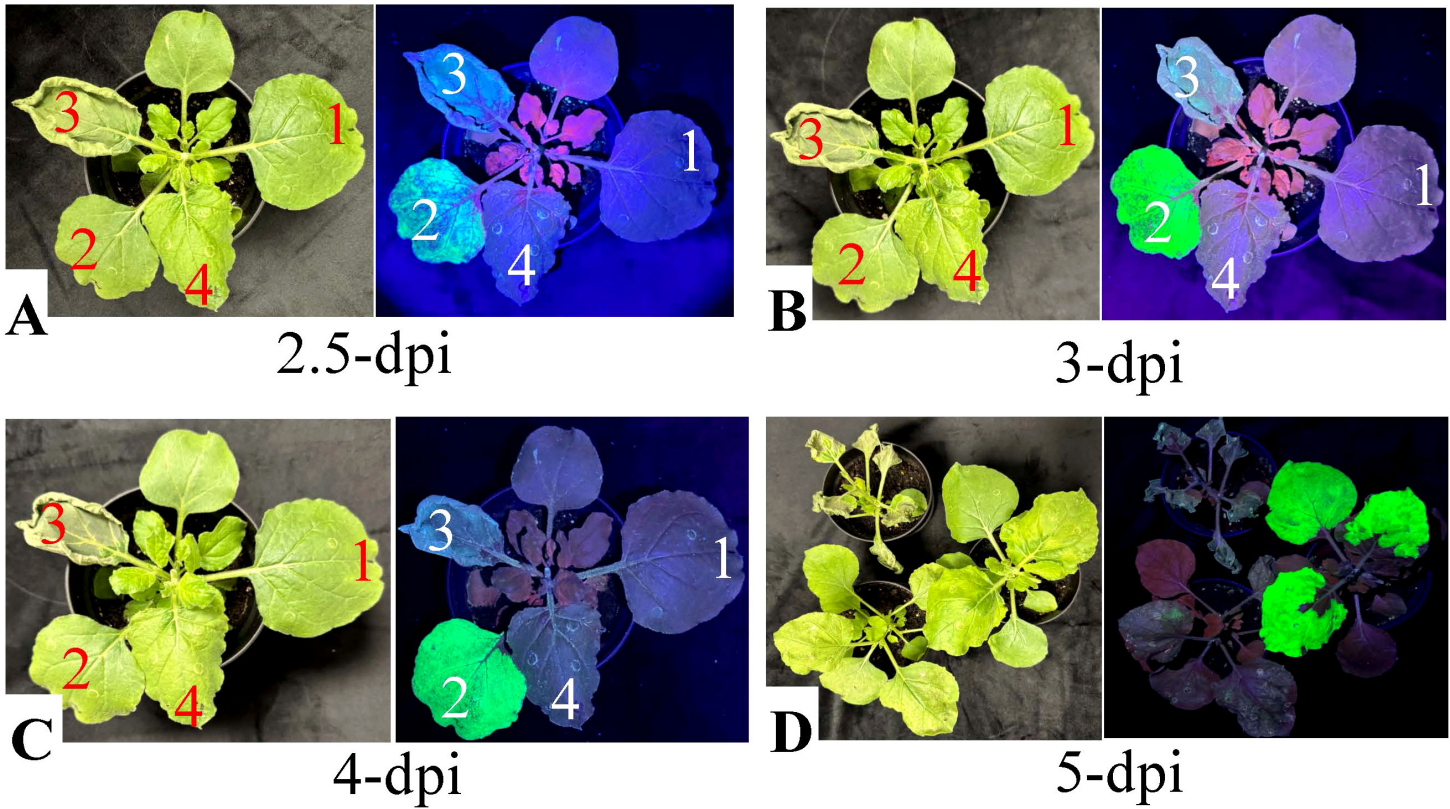
Evaluation of necrosis induced by nattokinase (NK) expression in *N. benthamiana* under normal and UV light. Fully expanded *N. benthamiana* leaves were infiltrated with *A.tumefaciens* strain EHA105 (1, negative control), or EHA105 carrying the vectors pBY!GFP (2), pBY!fNatto (3, full-length nattokinase), or pBY!mNatto (4, mature-length nattokinase). Plants were maintained under controlled growth conditions, and necrotic symptoms were documented at various time points. **(A)** Leaf images at 2.5 dpi under normal light. **(B)** Leaf images at 3 dpi. **(C)** Leaf images at 4 dpi under normal light. **(D)** Whole-plant images for treatments (2–4) captured under both normal and UV light at 5 dpi. Visible necrosis was evident under normal light, while UV imaging revealed reduced chlorophyll autofluorescence in necrotic areas. Leaves expressing full-length NK (pBY!fNatto) exhibited earlier onset and more severe necrosis compared to those expressing the mature form (pBY!mNatto), highlighting the greater cytotoxicity associated with full-length nattokinase expression.

The transient expression of full-length nattokinase in *N. benthamiana* resulted in pronounced necrosis and significant loss of tissue integrity. As shown in **Figure 2**, leaves infiltrated with the pBY!fNatto vector began showing abrupt stress responses at 2.5 dpi, coinciding with the onset of BeYDV-driven gene expression (as indicated by the onset of GFP fluorescence in **Figure 2A**). Early symptoms included yellowing, watery lesions, and marginal curling—classic indicators of early necrotic onset. Under UV light, the affected regions appeared gray, signifying chlorophyll degradation and early cell death.

By 3 dpi, necrosis intensified. Infiltrated areas became brittle and desiccated, suggesting a substantial loss of turgor pressure and potential vascular collapse (**Figure 2B**). Other observable features included pale gray-green discoloration, leaf shrinkage, and curling at the edges, consistent with oxidative damage and membrane rupture. By 4 dpi, the damage had progressed to advanced necrosis. Leaves exhibited widespread dark gray discoloration, severe wilting, a dry, papery texture, and clear signs of tissue collapse (**Figure 2C**). These observations confirm the strong cytotoxic effect of full-length nattokinase expression in plant tissue, indicating that it actively induces cell death when overexpressed in plant-based expression systems.

In contrast, leaves infiltrated with pBY!GFP, pBY!mNatto (mature-length nattokinase), or *Agrobacterium* with no T-DNA remained visually healthy through 4 dpi, with no observable necrosis. These findings further support that the necrotic phenotype is specifically associated with the expression of the full-length nattokinase construct.

By 5 dpi, UV fluorescence imaging revealed gray patches in pBY!mNatto-infiltrated leaves, indicating delayed chlorophyll degradation and tissue damage (**Figure 2D**). The affected tissues exhibited a mosaic pattern of translucent and yellow lesions, consistent with progressive cellular degeneration associated with mature nattokinase expression. Mild yellowing was also occasionally observed in pBY!GFP-infiltrated controls, likely reflecting background stress from BeYDV vector replication, although no obvious necrotic symptoms were detected.

### Full-length nattokinase expression significantly increases electrolyte leakage

**Figure 3:**
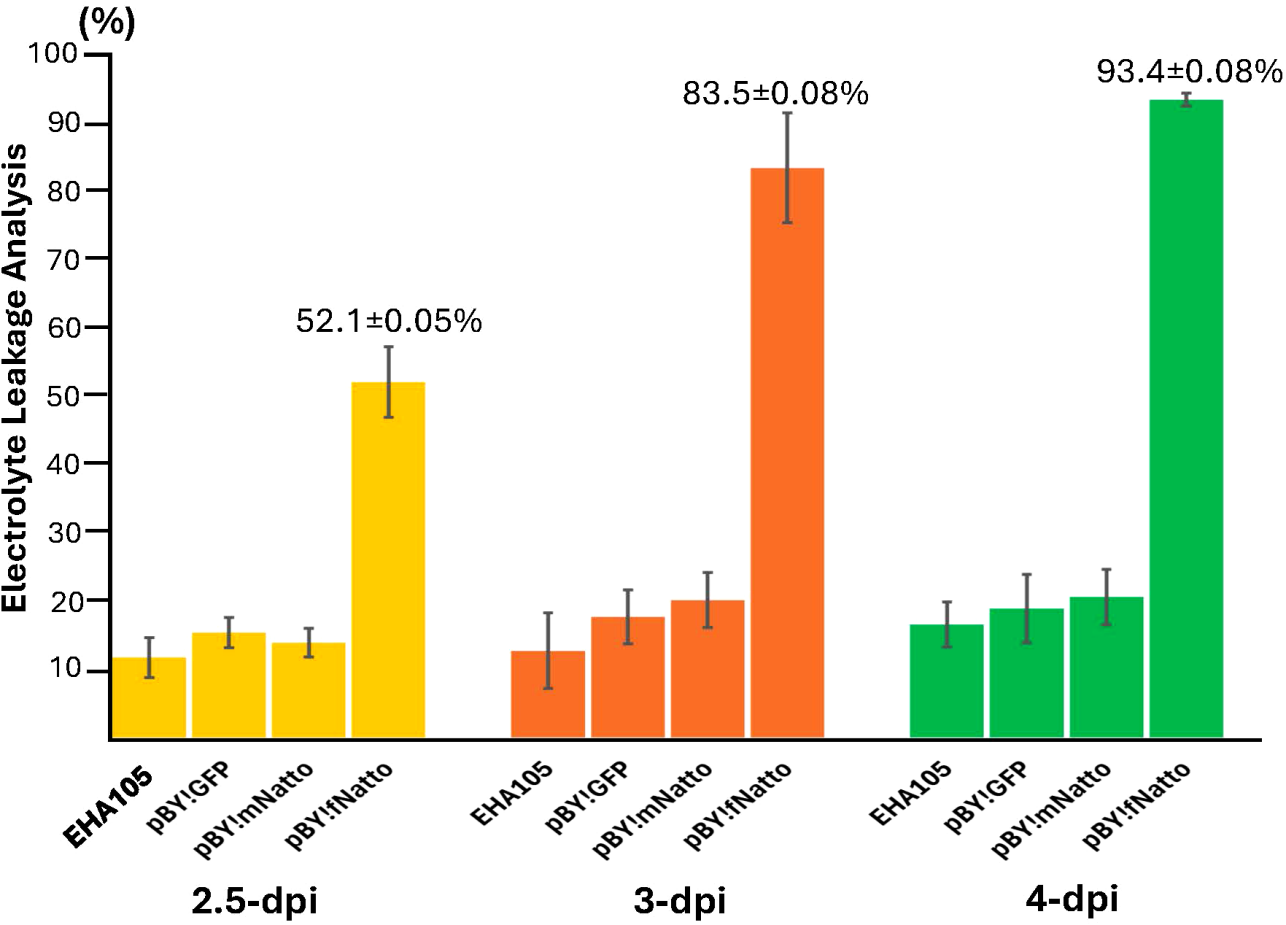
Electrolyte leakage (EL) analysis reveals significant tissue necrosis induced by Full-Length nattokinase Expression. Leaf discs were collected from *N, benthamiana* leaves infiltrated with pBY!fNatto (full-length nattokinase), pBY!mNatto (mature-length nattokinase), pBY!GFP (pBY), and *A. tumefaciens* strain EHA105 along (negative control). Samples were harvested at 2.5, 3, and 4 days post-infiltration (dpi). Conductivity was measured before and after heating, and EL percentages were calculated as the proportion of total electrolytes released prior to boiling. The results, presented as means ± SE (*n* = 3), show a significant (p < 0.0001) and progressive increase in electrolyte leakage in pBY!fNatto-treated samples compared to all control groups. Statistical analysis using pairwise Student’s *t*-tests confirmed highly significant differences at each time point, indicating that expression of full-length nattokinase induces substantial loss of membrane integrity and cell viability.

Electrolyte leakage (EL), a proxy for cell death due to loss of membrane integrity, was measured to quantify tissue damage following transient expression of nattokinase. As shown in **Figure 3**, leaves infiltrated with pBY!fNatto exhibited a 52.1% loss of membrane integrity by 2.5 dpi, indicating significant early cell damage (p < 0.0001) compared to all control treatments (EHA105, pBY!GFP, and pBY!mNatto), which showed no statistically significant differences among themselves (p > 0.1). By 3 dpi, EL in pBY!fNatto-treated samples rose sharply to 83.5%, and by 4 dpi, it reached 93.4%, consistent with extensive tissue necrosis (p < 0.0001 at both time points). In contrast, pBY!GFP, pBY!mNatto, and EHA105 controls consistently maintained low EL levels across all time points, with no significant variation (p > 0.1). These results confirm that the observed loss of membrane integrity is specifically linked to the expression of full-length nattokinase, highlighting its cytotoxic effect in plant tissues.

Due to the rapid and severe necrosis caused by full-length nattokinase expression, all subsequent experiments in this study focused on the mature form, pBY!mNatto.

### SDS-PAGE and western blot analysis of recombinant nattokinase

**Figure 4:**
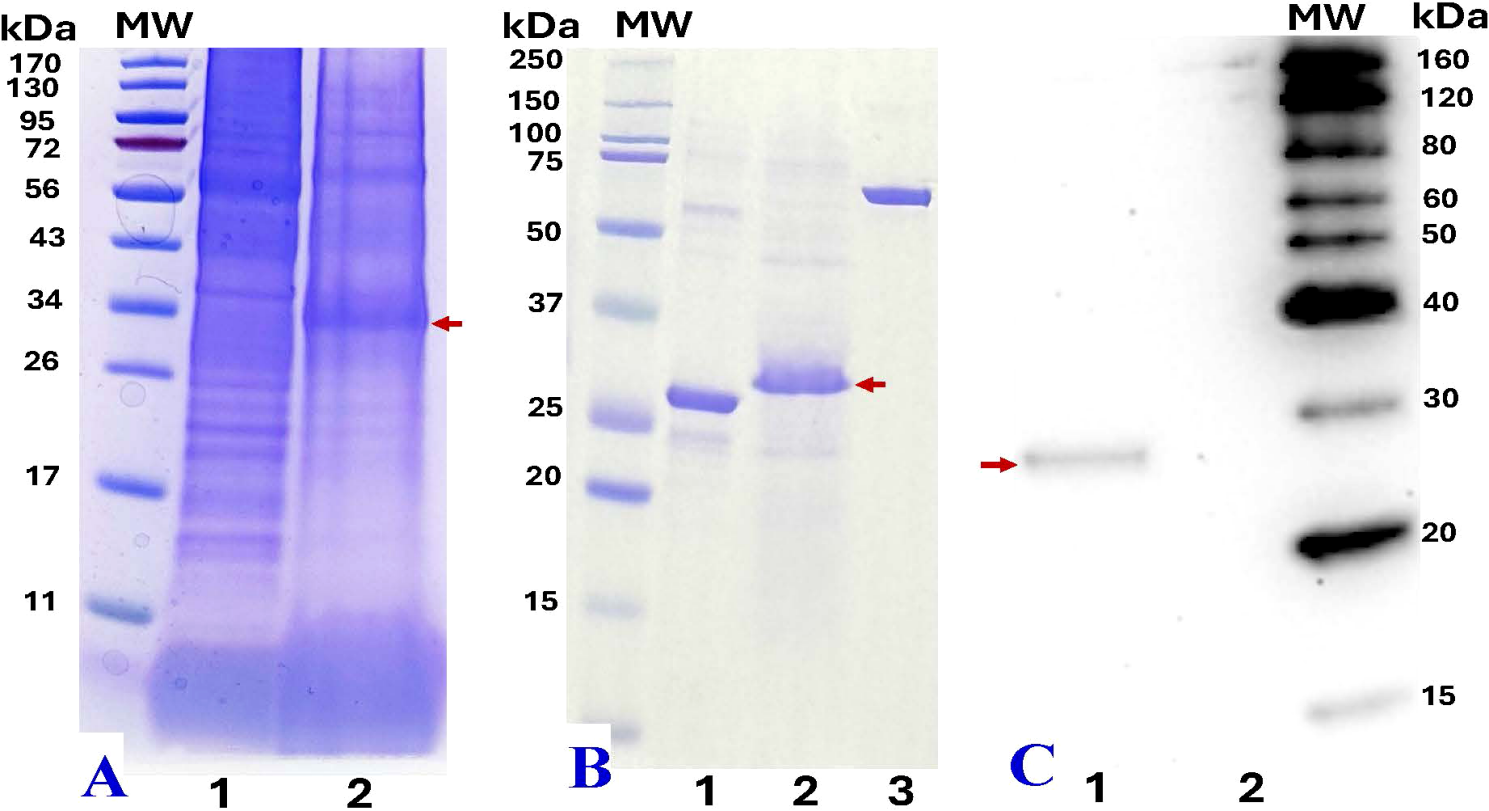
SDS-PAGE and western blot analysis of recombinant nattokinase (rNK). **(A)** SDS-PAGE analysis of crude protein extracts (20 µL) from wild-type leaves (Lane 1) and *N. benthamiana* leaves infiltrated with pBY!mNatto (mature-length nattokinase) at 4 dpi (Lane 2).**(B)** SDS-PAGE comparison of commercial nattokinase (3 µg, positive control, ~27.7 kDa, Lane 1), 6xHis-tag purified plant-derived rNK (~28.4 kDa, 10 µL, Lane 2), and BSA (2 µg, internal standard for ImageJ quantification, Lane 3). **(C)** Western blot analysis using anti-His probe detection: purified rNK (Lane 1) and His-tag eluate from non-infiltrated (wild-type) leaves (Lane 2). Molecular weight (MW) markers are shown alongside each gel for reference. Red arrows indicate the expected rNK protein bands at at ~28.4 kDa.

The total protein content from treated leaf areas was analyzed using SDS-PAGE and compared to the total soluble proteins from wild-type (WT) leaf tissue. At 4 dpi, leaves infiltrated with pBY!mNatto displayed a distinct ~28.4 kDa band (with C-terminal 6xHis tag) on SDS-PAGE gel (**Figures 4A, B**, Lane 2), consistent with the expected size of recombinant nattokinase (rNK). The band aligned with the predicted molecular weight of the nattokinase (NK) protein (27.7 kDa without his tag; **Figure 4B**, Lane 1). Western blotting using anti-His probe further confirmed the presence of rNK, showing a single band at ~28 kDa (**Figure 4C**). (**Figure 4C**). These findings confirm the successful expression of recombinant NK in the samples. The yield of NK was estimated to be approximately 370 µg/g leaf fresh weight (LFW), calculated using standard BSA as a reference and quantified with ImageJ software.

### Measure the enzymatic activity of nattokinase by casein and fibrin plate assay

**Figure 5:**
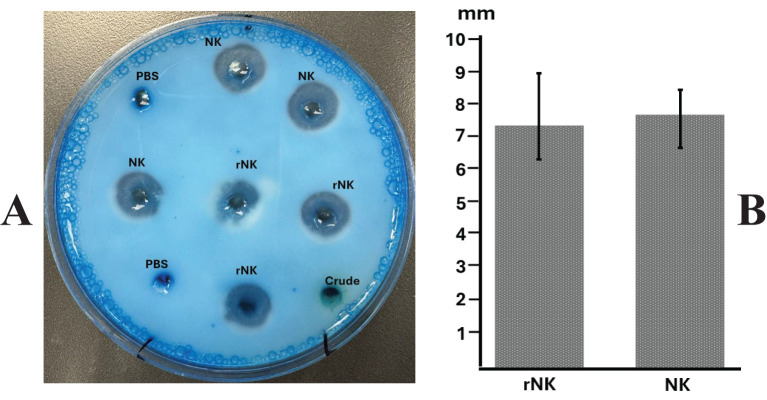
Assay of nattokinase activity on casein plate. **(A)** Representative image showing the hydrolysis halos produced by Nattokinase. **(B)** Quantification of lysis halo diameters (well diameter subtracted). PBS, Phosphate Buffered Saline, negative control; Crude, crude supernatant from wild-type leaves; NK: standard Nattokinase (AbMore BioScience Inc., Houston, TX, USA; Batch#: M2121-202401; Activity data:22,500 FU/g); rNK: plant-derived recombinant Nattokinase. Results show no significant difference between NK and rNK (P = 0.635, paired t-test, n = 3).

The casein plate assay (**Figure 5**) demonstrated that recombinant nattokinase effectively hydrolyzed casein, forming clear halos around the wells (**Figure 5A**). The mean diameters of the halos, calculated after subtracting the well diameter (5 mm), confirmed the proteolytic activity of the recombinant enzyme (**Figure 5B**). Statistical analysis showed no significant difference between recombinant nattokinase and the standard enzyme used as a positive control (P = 0.635, paired t-test). Negative controls, including PBS, exhibited no halo formation, further validating the specificity of nattokinase’s protease activity. These results highlight the ability of plant-derived recombinant nattokinase to efficiently hydrolyze casein, consistent with its known enzymatic properties.

**Figure 6:**
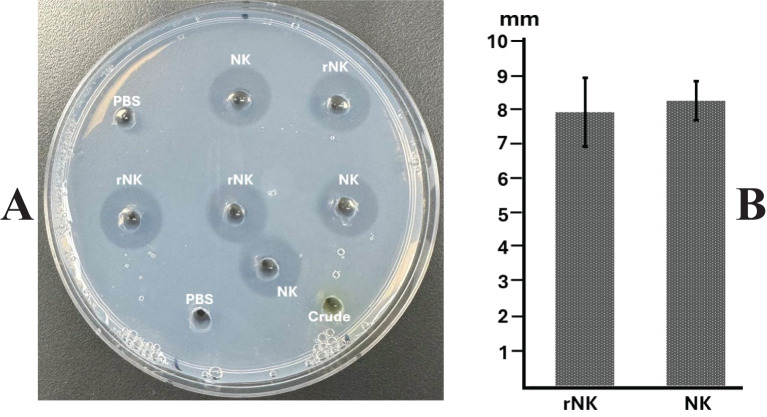
Assay of nattokinase activity on fibrin plate. **(A)** Representative image displaying the hydrolysis halos produced by Nattokinase. **(B)** Quantification of lysis halo diameters (well diameter subtracted). PBS, Phosphate Buffered Saline, negative control; Crude, crude supernatant from wild-type leaves; NK: standard Nattokinase (AbMore BioScience Inc., Houston, TX, USA; Batch#: M2121-202401; Activity data:22,500 FU/g); rNK: plant-derived recombinant Nattokinase. Results indicate no significant difference in activity between NK and rNK (P = 0.643, paired t-test, n = 3).

The fibrin plate assay (**Figure 6**) further demonstrated the fibrinolytic potential of recombinant nattokinase, providing a visual confirmation of enzyme activity through the formation of clear halos where fibrin was dissolved (**Figure 6A**). The diameters of the halos were measured to quantify fibrinolytic activity (**Figure 6B**). The results indicated no significant difference between the fibrinolytic activity of plant-derived recombinant nattokinase and the standard enzyme (P = 0.643, paired t-test). This suggests that the fibrinolytic potential of the plant-derived enzyme is comparable to that of the commercial standard reagent, which has a documented fibrinolytic activity of 22,500 FU/g. The comparable halo sizes validate the efficacy of using plant systems for producing functional nattokinase with therapeutic potential equivalent to commercially available products.

## Discussion

Plants—particularly *N. benthamiana*—have demonstrated significant potential as platforms for heterologous protein production (Buyel, 2024; Golubova et al., 2024; Kopertekh, 2024; Akher et al., 2025; Shanmugaraj et al., 2025). In this study, both the full-length and mature forms of nattokinase were transiently expressed in *N. benthamiana* to evaluate the plant-based system’s ability to produce and process this fibrinolytic enzyme originally derived from *B. subtilis*.

Previous reports have shown successful expression of full-length nattokinase in transgenic cucumber (Ni et al., 2023) and through transient expression in melon (Han et al., 2015), with confirmed thrombolytic activity. Although full-length nattokinase (pre-pro form) is a zymogen—requiring signal peptide and propeptide cleavage for activation—these findings suggest that some plant species may possess endogenous proteases capable of processing the enzyme into its mature, active form.

The construct pBY!GFP, used as a control in this study, directed GFP to ER via the amylase signal peptide (Diamos and Mason, 2019). In parallel, previous studies have demonstrated that the LPH signal peptide promotes efficient ER targeting, secretion, and proper folding of recombinant proteins—including immunotoxins and GFP reporters—when transiently expressed in *N. benthamiana* (Knödler et al., 2023). In these studies, ER-targeted GFP expression did not induce visible tissue damage or necrosis, suggesting that ER localization itself is not intrinsically cytotoxic.

In contrast, BeYDV-driven expression of full-length nattokinase (pBY!fNatto) in *N. benthamiana* led to rapid and severe tissue necrosis. Necrotic symptoms appeared as early as 2.5 dpi (**Figure 2A**), corresponding with the onset of BeYDV-mediated gene expression (Diamos and Mason, 2019). By 3 dpi, extensive tissue collapse and desiccation were observed (**Figure 2B**), indicating proteolytic activity. Electrolyte leakage assays confirmed 83.5% loss of membrane integrity (**Figure 3**), highlighting the cytotoxic effects of unregulated protease activity in planta.

Previous studies have shown that the inclusion of a secretion signal peptide can significantly influence the subcellular localization and accumulation levels of recombinant proteins in plants. Targeting proteins to organelles such as the endoplasmic reticulum (ER) typically enhances folding, stability, and yield, whereas omission of the signal peptide often results in cytosolic retention, leading to increased susceptibility to misfolding and proteolytic degradation, ultimately reducing expression and activity (Liu and Timko, 2022). In this study, the LPH signal peptide was employed to direct recombinant nattokinase into the ER, facilitating its entry into the secretory pathway and supporting improved protein stability and accumulation (Voss et al., 1995; Vaquero et al., 1999; Schiermeyer et al., 2005; Knödler et al., 2023). However, in the case of the full-length construct, the combination of ER targeting via the LPH peptide and high-level expression driven by the BeYDV replicon likely induced acute ER stress. This stress was presumably caused by the accumulation of unfolded or misfolded proteins, which overwhelmed the plant’s protein folding and quality control machinery (Wan and Jiang, 2016; Buono et al., 2019).

Improper full-length signal peptide or propeptide processing and unregulated or mislocalized protease activity may have further disrupted cellular homeostasis, while immune responses such as the hypersensitive response (HR) likely amplified the damage, culminating in rapid programmed cell death (Buono et al., 2019). It is highly plausible that the presence of both the eukaryotic ER signal peptide (LPH) and the retained prokaryotic signal peptide in the full-length nattokinase led to abnormal membrane anchoring or mislocalization of the protease. This misrouting likely exacerbated ER stress and contributed to activate plant defense signaling and leads to rapid program cell death (PCD) (Manghwar and Li, 2022; Chen et al., 2023). These observations underscore the importance of removing native bacterial targeting sequences when designing constructs for expression in eukaryotic systems. Collectively, these findings suggest that full-length nattokinase is unsuitable for high-level transient expression in *N. benthamiana* due to its incompatibility with host cellular machinery and its strong cytotoxic effects.

Foreign proteases can trigger necrosis in plant tissues due to their unregulated activity, which may interfere with endogenous proteins critical for maintaining cellular structure and function. Our previous work demonstrated that transient expression of another protease, Lumbrokinase, caused severe necrosis in *Nicotiana tabacum*, with electrolyte leakage exceeding 80% by 4 dpi and a relatively low recombinant protease yield of 20 µg/g fresh weight (Dickey et al., 2017). Similarly, Ma et al. (2019) reported that transient expression of bioactive recombinant Reteplase in *N. benthamiana* using the pJL-TRBO-G vector resulted in pronounced necrosis by 7 dpi, coinciding with peak foreign protein accumulation. These findings suggest that protease interactions with native plant proteins may disrupt essential cellular functions, triggering a cascade of degradation events and stress responses. Such necrotic outcomes likely reflect a plant defense mechanism—intended to contain damage, limit water loss, and redirect metabolic resources to enhance survival under proteolytic stress.

In contrast, the mature form of nattokinase—lacking the native signal peptide and propeptide—proved significantly more compatible with plant expression. Leaves infiltrated with pBY!mNatto remained visibly healthy through 4 dpi (**Figure 2C**), with only mild tissue damage observed by 5 dpi (**Figure 2D**). This delayed onset of necrosis may reflect the slower accumulation and more controlled activity of the mature enzyme, resulting in a less acute proteolytic environment and reduced cytotoxic stress on plant tissues. Additionally, the use of a modified BeYDV system, which enables separate and regulated expression of the BeYDV replication proteins Rep and RepA, likely contributed to reduced cell death during mature nattokinase expression (Diamos and Mason, 2019).

Functionally, the plant-derived mature nattokinase retained its biochemical activity, as confirmed by casein hydrolysis and fibrin degradation assays. Clear zones on casein-containing plates indicated robust protease activity, while transparent halos in fibrin plates were comparable in size to those formed by commercial nattokinase, validating its fibrinolytic potential. These findings indicate that the recombinant enzyme expressed in *N. benthamiana* retains functional proteolytic activity, suggesting it achieved a conformation compatible with substrate recognition and catalytic function.

Importantly, this activity was achieved without inducing significant tissue necrosis during the early expression period, supporting the feasibility of using plants for the functional production of proteolytic enzymes like nattokinase. The ability to replicate its native function—degrading fibrin, the key protein in blood clots—positions plant-produced nattokinase as a promising candidate for therapeutic or nutraceutical applications.

## Conclusion

This study demonstrates that *N. benthamiana* is a viable host for the transient expression of biologically active mature nattokinase, while also revealing the severe cytotoxicity associated with overexpressing the full-length zymogen form. These findings highlight the importance of construct design in achieving functional and safe expression of nattokinase in plant systems. To enhance yield and reduce host tissue damage, future optimization strategies—such as foliar application of ascorbic acid to suppress leaf stress (Nosaki et al., 2021), compartment-specific targeting (Adam, 1996), or co-expression of protease inhibitors—may prove beneficial. Beyond whole-plant agroinfiltration, Nattokinase could be scaled using plant cell suspension cultures, which provide sterile and controlled environments conducive to consistent recombinant protein production. Suspension cultures derived from fast-growing lines such as tobacco BY-2 cells (Karki et al., 2021) can be genetically engineered for either stable or transient expression of Nattokinase. Additionally, recent innovations—such as cell pack technology (Gengenbach et al., 2020) —have enhanced the scalability, throughput, and reproducibility of transient expression workflows, further supporting the potential of plant platforms for industrial-scale biomanufacturing. Together, these strategies support the development of efficient plant-based production platforms for therapeutic and industrial-grade nattokinase.

## Data Availability

The datasets presented in this study can be found in online repositories. The names of the repository/repositories and accession number(s) can be found in the article/supplementary material.
